# Validation of a Rapid Host-Protein Score to Discriminate Bacterial from Viral Infections in Hospitalized Febrile Pediatric Patients

**DOI:** 10.3390/children12030381

**Published:** 2025-03-19

**Authors:** Maria Noni, Eleni Kalogera, Athina Xydia, Georgios Paradeisis, Kalliopi Spyridopoulou, Levantia Zachariadou, Evanthia Botsa

**Affiliations:** 1 First Department of Pediatrics, Medical School, National and Kapodistrian University of Athens, “Aghia Sophia” Children’s Hospital, 115 27 Athens, Greece; mnoni@med.uoa.gr; 2 Microbiology Department, “Aghia Sophia” Children’s Hospital, 115 27 Athens, Greece; kalogerael@yahoo.gr (E.K.); athinaxydia95@gmail.com (A.X.); gioparadisis@med.uoa.gr (G.P.); kaspyrido@gmail.com (K.S.); levantia@otenet.gr (L.Z.); 3 First Department of Pediatrics, “Aghia Sophia” Children’s Hospital, 115 27 Athens, Greece

**Keywords:** bacterial infection, co-infection, BV score, hospitalized patients, pediatrics

## Abstract

**Background:** The MeMed BV^®^ BV score is a novel, promising host-protein score, differentiating bacterial from viral infections, that combines the expression levels of tumor necrosis factor-related apoptosis-inducing ligand (TRAIL), interferon gamma-induced protein-10 (IP-10), and C-reactive protein (CRP). The aim of our study was to determine its diagnostic accuracy in hospitalized febrile children. **Methods:** A prospective study was performed from December 2023 to April 2024 in two pediatric clinics at “Aghia Sophia” Children’s Hospital, Athens, Greece. Patients > 3 months old, presenting with fever, clinical suspicion of acute infection, and symptoms onset up to 7 days prior were considered eligible. Patients with cancer, Human Immunodeficiency Virus (HIV), Hepatitis B Virus (HBV), Hepatitis C Virus (HCV), Tuberculosis (TB), and immunodeficiency were excluded. Two pediatricians reviewed the clinical, laboratory, microbiological, and radiological data and assigned the final diagnosis. The experts were blinded to the BV scores. **Results:** One hundred and thirty-five patients were enrolled. The predominant medical condition was respiratory tract infection (59.3% lower, 26.7% upper). Twenty-nine (21.5%) patients were diagnosed with bacterial infections. The BV score demonstrated a sensitivity of 96.2%, specificity of 88.7%, and negative predictive value (NPV) of 98.9% for bacterial infections. Equivocal BV scores were reported in 8.9% of cases and were excluded from calculations. The area under the curve was 0.96 (95% CI: 0.93–0.99). A ROC curve analysis was performed, and the optimal cut-off score was estimated at 60, providing a sensitivity of 93.1%, specificity of 88.7%, and NPV of 97.9%. **Conclusions:** In our study population, the BV score showed high sensitivity and NPV in bacterial infection diagnosis. Further studies are needed to assess the possibility of replacing the “equivocal” range with a narrower one or a specific cut-off value.

## 1. Introduction

Bacterial and viral infections are frequently clinically similar, which can result in inappropriate patient management and unnecessary use of antibiotics. Traditionally used host biomarkers, such as CRP and procalcitonin, often fail to discriminate between viral and bacterial infections in children. To enhance the performance of individual host proteins, combining multiple proteins into a single predictive score has been suggested. The MeMed BV^®^ (BV) score is a novel host-response technology developed for differentiating bacterial from viral infections [1]. The signature consists of the circulating levels of three immune proteins: C-reactive protein (CRP), tumor necrosis factor-related apoptosis-inducing ligand (TRAIL), and interferon gamma-induced protein-10 (IP-10).

These proteins exhibit differential expression in response to acute infections. TRAIL, a member of tumor necrosis factor family, plays an important role in regulating programmed cell death, a crucial mechanism in viral infections. Its expression increases during viral infections and decreases in bacterial infections. IP-10, a small cytokine involved in various cellular processes, such as chemotaxis and cell growth, is induced in response to bacterial infections and even more so in response to viral infections. CRP, an inflammatory marker, is upregulated in response to a variety of inflammation stimuli, including bacterial infections [1]. The algorithm integrates immune responses across multiple biological pathways, providing an accurate assessment of whether the host is responding to a bacterial or viral infection.

The test generates a numerical score, categorized into discrete interpretation bins that indicate the increasing likelihood of a bacterial infection. The score ranges from 0 to 100. There are thresholds for bacterial infections (or co-infection; 65 < score ≤ 100) and viral (or other non-bacterial; 0 ≤ score < 35) infections, as well as for ambiguous scores (35 ≥ score ≤ 65). Multiple clinical trials supported these criteria, such as a blinded analysis in children under 5 years old with fever of unknown origin and probable lower respiratory infection (RTI). The BV score achieved 91.1% specificity (95%CI: 87.9–93.6), 86.7% sensitivity (95%CI: 75.8–93.1), and 12.5% equivocal instances [2].

Its use has primarily been evaluated as a supportive tool at the emergency department (ED) in conjunction with clinical evaluations and further laboratory tests [3,4,5,6]. However, the BV score’s diagnostic accuracy for determining bacterial infections in hospitalized patients in the absence of a positive culture has not been studied extensively.

The aim of this study was to evaluate the use of BV scores in hospitalized Greek children with clinically suspected infections.

## 2. Materials and Methods

### 2.1. Study Design and Study Population

This prospective study was conducted from December 2023 to April 2024 in two pediatric clinics of “Aghia Sophia” Children’s Hospital, the largest pediatric tertiary care hospital in Greece. The BV score was performed on 135 hospitalized pediatric patients. All patients, aged 3 months to 16 years old, were admitted to the pediatric ward due to fever.

The inclusion criteria were febrile respiratory infection or fever of unknown origin. Patients with a fever for up to seven days prior to hospital admission were included. Patients with a medical history of active malignancy, Human Immunodeficiency Virus (HIV), Hepatitis C virus (HCV), Hepatitis B Virus (HBV), Tuberculosis (TB), or other primary or secondary immunodeficiency, as well as those who had received antibiotic treatment for more than two days prior to admission were excluded, based on the manufacturer’s instructions for the test’s performance.

### 2.2. Data Collection and Study Procedures

All participants underwent a standard work-up, including demographic characteristics, medical history, and physical examination, laboratory evaluations, with complete blood cell counting, biochemistry, blood gas, procalcitonin, and microbiologicaltesting, including cultures from various biological samples. Chest X-rays or chest computed tomography were performed when necessary.

Νasopharyngeal swabs were collected for multiplex polymerase chain reaction (PCR) testing for upper respiratory infections from all patients. The nasopharyngeal swab samples (COPAN UTM system, COPAN Diagnostics, Murrieta, CA, USA) were subjected to BIOFIRE^®^ Respiratory 2.1 plus FilmArray^®^ Panel, BioMérieux^®^, Marcy-l’Étoile, France.

Concurrently with routine blood sampling, serum samples for BV measurements were collected from all patients, according to the manufacturer’s instructions. The BV score was calculated by using TRAIL, IP-10, and CRP measurements in the ImmunoXpert™ software (CDSS) (MeMed, São Paulo, Brazil), using the MeMed Key^®^ to generate a score between 0–100. The blood for study measurements was processed within two hours. Simultaneously, CRP was measured by the nephelometry method (Beckman Coulter) (CRPn). According to the manufacturer’s instructions, score cut-offs were at <35 for viral diseases and >65 for bacterial infection, with scores between 35 and 65 deemed equivocal. The BV scores were recorded separately, accessible only to infectious disease specialists. The clinicians performing the BV score assessments were not provided with the patients’ clinical or diagnostic status. Two pediatricians reviewed the clinical, laboratory, microbiological, and radiological data and assigned a diagnostic label: bacterial (including bacterial and viral co-infection) or viral infection. The experts were blinded to the BV score. Thus, the pediatricians’ decisions were multifactorial. The diagnosis of bacterial infections was established based on a combination of clinical judgment, supportive laboratory and imaging findings, and not only on culture-positive results, as culture-negative results are common in everyday medical practice. Clinical signs and symptoms characteristics of bacterial infections (e.g., focal pulmonary findings consistent with pneumonia and pain consistent with sinusitis), laboratory markers, including elevated inflammatory markers, such as high CRP levels, procalcitonin, or white blood cell count, suggestive of a bacterial etiology in the context of the patient’s illness, radiological findings (e.g., an infiltrate on chest X-ray indicative of bacterial pneumonia), other diagnostic tests consistent with bacterial infections (e.g., positive multiplex bacterial PCR), and finally, a clinical course and response to antibiotic treatment were considered prerequisites for the diagnosis of bacterial infections.

Using these criteria, an expert assessment was made for each case. This approach ensured that even if a pathogen could not be cultured, patients with a high probability of bacterial infection (based on the above parameters) were appropriately classified.

Our aim was to evaluate the use of the BV score in hospitalized Greek children with clinically suspected infections and to determine the optimal cut-off value for discriminating viral from bacterial infections.

### 2.3. Statistical Analysis

Descriptive statistics were completed or all variables. The categorical data were expressed as absolute numbers and proportions (%). The continuous data were tested for normality using statistical tests (Kolmogorov–Smirnoff test) and graphical methods (histogram and Q–Q plot). Normally distributed variables were reported as the mean and standard deviation (SD), while skewed variables as the median and interquartile range (IQR). For normally distributed variables, the Student’s t-test was applied to assess differences between two groups, whereas, for skewed variables, the Mann–Whitney U test was performed. For categorical data, we performed Chi-square tests for comparisons, or Fisher’s exact tests if data were not suitable for Chi-square testing.

Specificity, sensitivity, positive predictive value (PPV), negative predictive value (NPV), positive likelihood ratio (LR+), negative likelihood ratio (LR−), and the respective 95% confidence intervals (CIs) were then calculated based on two predefined score thresholds. Cases with scores between 35 and 65 were classified as equivocal, excluded from these calculations, and presented as a separate rate.

ROC curve analysis was conducted to assess the performance of the BV score without excluding equivocal patients. The area under the curve (AUC) was used to quantify the performance, and the optimal cut-off value was determined using Youden’s index: sensitivity + specificity − 1.

The statistical analyses were carried out using the PSAW Statistics 21 (SPSS, Chicago, IL, USA, http://www.spss.com). The statistical significance was set at *p* < 0.05.

## 3. Results

### 3.1. Study Population Characteristics

During the study period (December 2023 to April 2024), 2324 patients were hospitalized in two pediatric clinics that participated in the study. One hundred and thirty-five patients met the inclusion criteria. Eighty (59.3%) patients were male, and the median age was 3.5 years old (IQR 1.3–6.0). Nineteen (14.1%) patients were infants less than 12 months of age. The median duration of fever before admission was 2 days (IQR 1–3.3), and the median duration of hospitalization was 3.5 days (IQR 2–6).

One hundred and sixteen (85.9%) patients were diagnosed with respiratory tract infections: 36 (26.7%) with upper respiratory tract infections (URTIs) and 80 (59.3%)with lower respiratory tract infections (LRTIs). The remaining patients were diagnosed with urinary tract infection (UTI) (*n* = 4, 3%) or another type of infection (*n* = 15, 11%).

Twenty-nine (21.5%) patients had a final diagnosis of bacterial infection. The most common bacterial diagnosis was LRTI (16 patients; 55%), followed by URTI (5 patients; 17%), UTI (4 patients; 14%), and bacteremia (4 patients; 14%). Sixty-one (45.2%) patients received antibiotic treatment during hospitalization.

As anticipated, the hospitalization duration was significantly shorter for patients suffering from viral infections compared to those with bacterial infections (3 vs. 5 days, respectively; *p*-value = 0.005). Furthermore, patients with viral infections were significantly younger (3 vs. 6 years respectively; *p*-value ≤ 0.001). Antibiotics were prescribed to 32 (30.2%) patients with viral infections and to all patients with bacterial infections.

Among the patients diagnosed with URTIs or LRTIs, no significant differences were reported in the median age and duration of fever before admission. However, patients with LRTIs had a longer median duration of hospitalization (*p* = 0.04) and a higher percentage of antibiotic consumption (*p* = 0.05).

Molecular multiplex-nested-PCR testing achieved a high positivity rate of 83% in our study group. The most commonly detected viruses were Human rhinovirus/enterovirus, RSV, and Influenza virus. A virus was found in the respiratory systems in 19 (65.5%) of the patients with bacterial infections (Table 1).

### 3.2. BV Score Performance

**Figure 1:**
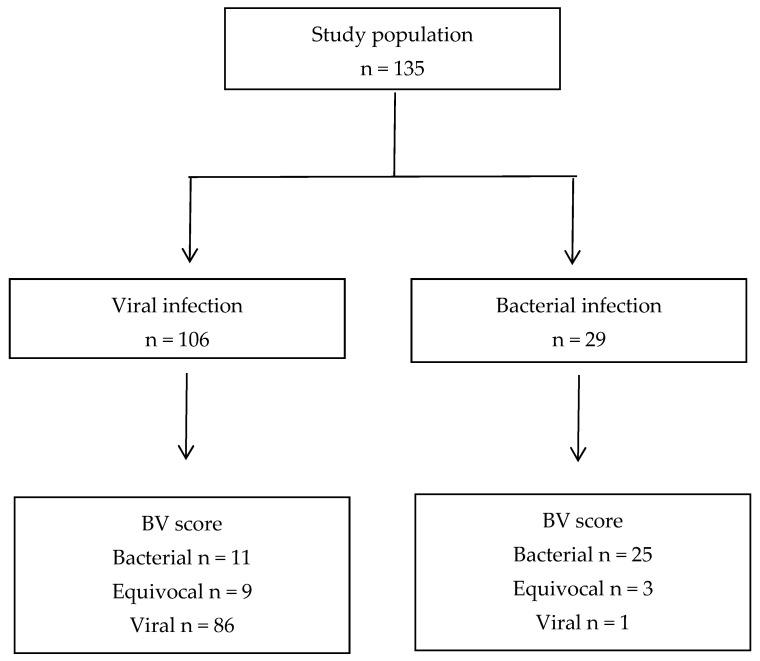
Patient enrollment flow.

Eighty-seven (64.4%) patients had a BV score <35. Eighty-six (98.9%) of them were diagnosed with viral infections, and only one (1.1%) had a bacterial infection. This patient was diagnosed with an LRTI caused by *Chlamydia pneumoniae*. In 12 (8.9%) cases, the BV score was equivocal. Of these, three patients were diagnosed with bacterial infections (two with streptococcal pharyngitis and one with LRTI). Additionally, 36 (26.7%) patients had BV scores > 65. Among them, twenty-five (69.4%) were diagnosed with bacterial infections, while 11 (30.6%) patients had a viral infection (Figure 1).

Among the patients diagnosed with URTIs or LRTIs, no significant differences were observed in the percentage of BV scores falling within the bacterial or viral range.

The BV score offered a sensitivity of 96.2% (95% CI: 80.4–99.9%), specificity of 88.7% (95% CI: 80.6–94.2%), PPV of 69.4% (95% CI: 56.5–79.9%), NPV of 98.9% (95% CI: 92.6–99.8%), LR+ of 8.5 (95% CI: 4.8–14.9), and LR− of 0.04 (95% CI: 0.01–0.30). The BV score was performed with an AUC of 0.962 (95% CI: 0.931–0.993), with a *p*-value < 0.001.

**Figure 2:**
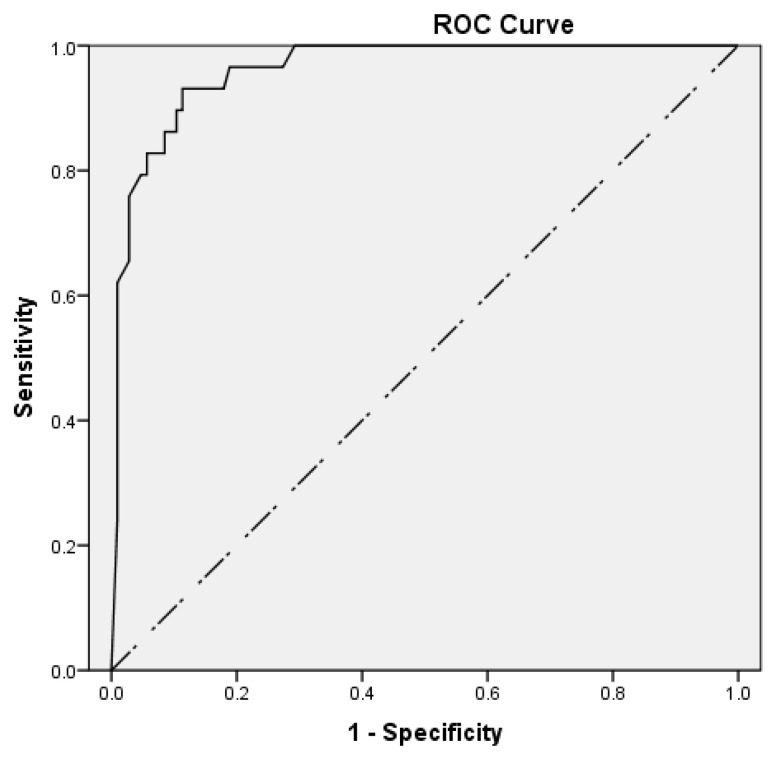
ROC curve analysis showing the performance of the BV score.

According to the ROC curve analysis, the best cut-off value was estimated at 60 and provided 93.1% sensitivity, 88.7% specificity, 69.2% PPV, and 97.9% NPV (Figure 2).

### 3.3. Comparison to Routine Biomarkers

The BV score outperformed routine biomarkers, including CRPn, white blood cells (WBCs), and absolute neutrophil count (ANC) (Table 2). The CRPn at a cut-off of 60 mg/L exhibited the same sensitivity and NPV as the BV cut-off at 60. However, the CRPn showed lower specificity and PPV. In contrast, both the WBCs and ANC, exhibited lower sensitivity, specificity, PPV, and NPV compared to the BV score when cut-off values were set at 15,000/μL and 10,000/μL, respectively. Regarding procalcitonin, it was excluded from the final analysis due to its unavailability for the entire study period, resulting in numerous missing values.

### 3.4. Estimation of the Impact of the BV Score on Antibiotic Use

The current antibiotic prescribing practice, as documented in medical records, was compared to a hypothetical scenario incorporating the BV score. This comparison was based on the assumption that a timely, contraindicating BV score would have led to a change in antibiotic management. Given that antibiotics were prescribed to 32 (30.2%) patients with viral infections, it was estimated that integrating the BV score could potentially reduce overall antibiotic prescriptions from 45.2% to 21.5%. As previously noted, antibiotics were prescribed to 61 (45.2%) patients, including all patients with bacterial infections (*n* = 29) and 32 patients with viral infections. Among these 32 patients, 29 had BV scores within the viral range, two had equivocal BV scores, and one had a BV score within the bacterial range. Taking into consideration only patients with viral infections whose BV scores fell within the viral range, it was estimated that BV scores could potentially reduce antibiotic prescriptions from 45.2% to 23.7%.

## 4. Discussion

This is the first study to evaluate the use of the BV score in hospitalized febrile children aged >3 months with clinically suspected infections. To date, the BV score has primarily been assessed in populations of relatively low severity, including febrile patients presenting to emergency departments or urgent care centers. The novelty of this study lies in its focus on hospitalized patients who typically present with more severe symptoms, including difficulties with feeding or breathing, dehydration, weakness, and exhaustion. This study establishes the high diagnostic accuracy of the BV score in differentiating bacterial from viral infections in hospitalized patients experiencing a higher severity of illness. The strengths of the study include a robust design and the collection of comprehensive, wide-ranging patient data to support the final diagnosis, which was assigned by two pediatricians. Additionally, the diverse study population encompasses patients with varying comorbidities and multiple pathogens. As the largest tertiary hospital in the Balkans, our institution further supports the broader applicability of these results. Recent reports from the manufacturer indicate that the BV score results will be available within 15 min from a small serum or whole blood sample. This will expand the utility of the BV score in settings that require rapid diagnostic turnaround, such as emergency departments, intensive care units, etc.

In our Greek study population, the BV score attained a sensitivity of 96.2% and specificity of 88.7%, comparable to that reported in previous studies in children [3,7]. The BV score accurately classified over 91.1% of our patients as having either bacterial (score > 65) or viral (score < 35) infections. The remaining 12 (8.9%) cases were categorized as “equivocal” (35 ≤ score ≤ 65), a percentage comparable to other published studies [2]. Among these equivocal cases, three patients were diagnosed with bacterial infections (two with streptococcal pharyngitis and one with LRTI), with scores ranging from 52 to 65. The remaining patients, diagnosed with viral infections, had scores between 52 and 63 and upper respiratory multiplex PCR revealed pathogens, such as Human rhinovirus/enterovirus, RSV, Coronavirus, and Parainfluenza.

Of note, an equivocal test result does not provide etiological information, and physicians are advised to decide considering other available clinical data. Therefore, these results were excluded from the calculation of sensitivity and specificity. However, according to ROC curve analysis, which included patients with equivocal scores, the BV score exhibited high diagnostic accuracy. The optimal cut-off value was estimated at 60, which lies within the “equivocal” range and is near its upper limit. Further studies in larger populations are necessary to assess the possibility of replacing the “equivocal” range with a narrower one or a specific cut-off value so as to enhance the test’s clinical applicability.

Among our study cohort, only one false negative result was identified. This patient, diagnosed with atypical pneumonia caused by *Chlamydia pneumoniae*, had a low BV score of <35. According to Papan et al., children with atypical pneumonia caused by *Mycoplasma pneumoniae* had low, viral-like BV scores. This could be attributed to significantly elevated TRAIL levels, resembling those found in viral infections and clearly indicating a distinctive immune response in atypical pneumonia [8]. Our patients also demonstrated high TRAIL levels. As these observations seem to underscore the complementary role of the BV score in atypical pneumonia, further studies in larger populations are necessary in order to study the improvement of the BV score’s diagnostic accuracy in cases of atypical pneumonia. The diagnosis of atypical pneumonia is quite challenging, mainly due to the intrinsic characteristics of the organisms which appear to be intermediate between bacteria and viruses. Until now, a combination of clinical features and biomarkers seems to be more helpful for the early diagnosis of atypical pneumonia to avoid the use of ineffective first-line empirical β-lactam antibiotics and enable the evaluation of targeted treatment in severe cases [9].

According to our results, leukocytosis was demonstrated to be a poor indicator of bacterial versus viral infection, a conclusion similarly reached for the ANC. The CRP performed better than the WBCs and ANC, with its results closely aligned with those of the BV score. This outcome was expected, as the BV score combines the expression levels of TRAIL, IP-10, and CRP [10,11]. Traditional biomarkers, such as WBCs, CRP, and procalcitonin, often fail to discriminate viral and bacterial infections in children. Elevated CRP levels are suggestive of a bacterial infection but may be observed in patients with certain viral strains (e.g., adenovirus and influenza) and non-infectious conditions [12]. Regarding procalcitonin, it may be elevated not only in bacterial pneumonia but also in some uncomplicated viral infections, such as influenza or SARS-CoV-2, due to an increased inflammatory response [13]. Presepsin, a relatively new biomarker, has been proposed for diagnosis in neonatal sepsis, however, its role in other clinical scenarios, such as RTI, is less studied [14]. The lack of a definitive diagnostic test continues to hinder early diagnosis and appropriate management in pediatric care. In recent years, new methods have been studied to support clinicians in differentiating viral from bacterial infections. Transcript host RNA signatures from host leukocytes in response to a clinically undifferentiated infection are a new promising tool, but they have not been completely established in the pediatric population. One of the challenges hindering the clinical application of transcriptomics is its high cost and the requirement for specialized skills to interpret results. Technical advances are on the way, such as platforms that are able to allow transcriptomic analysis at a single-cell resolution [15]. Circulating host proteins offer a promising alternative tool, as they can be quickly measured at the point of care. To enhance their diagnostic performance, combining several proteins into a single predictive score has been proposed. Recent studies have suggested that integrating unrelated host proteins involved in different pathways, such as BV scores, may improve diagnostic accuracy. In particular, incorporating newly identified host proteins that are upregulated in viral infections could serve as an innovative complement to bacterially induced proteins currently used in clinical use [1]. A potential limitation of this study is the relatively small number of confirmed bacterial cases (29/135 = 21.5%). However, this is consistent with the low incidence of bacterial infections in the pediatric population compared to adults. We did not include immunosuppressed patients or infants under 3 months of age, for whom an adjunctive test could be helpful. Moreover, the BV scores were not shared with attending physicians, which limited our ability to directly evaluate their impact on antibiotic prescribing practices. Despite this, we estimated that the BV score could reduce antibiotic prescriptions from 45.2% to 23.7%, an important finding, although based on retrospective analysis. Future studies should validate this reduction in clinical practice. We should mention that only cases with BV scores within the viral range contributed to this estimation. In practice, clinicians may be reluctant to withhold antibiotics in cases with equivocal results due to diagnostic uncertainty. Thus, narrowing the “equivocal” range or setting a more specific cut-off value, as we have already mentioned before, could have a further impact on antibiotic prescription. A pilot study performed by Kalmovich et al. showed that the BV score had a significant impact on clinical decision making, with physicians prescribing antibiotics in accordance with the BV score results in 81.7% of all cases. In total, they reported that the BV score supported or altered their decision making in 87.0% of cases [5]. Future prospective interventional studies should be designed where the BV score result is actively shared with clinicians during patient management. Thus, researchers could directly observe how the availability of the BV score influences diagnostic confidence, antibiotic prescribing behavior, and patient care. Such studies will help determine the real-world impact of the BV assay and improve patient outcomes.

To conclude, the BV score accurately differentiated viral from bacterial infections in our study population. As a biomarker, it has the potential to serve as a valuable aid in clinical decision making, including antibiotic treatment, hospitalization duration, and further laboratory evaluation. Additional studies are needed to refine the cut-off values and expand the clinical applicability of the BV score, particularly regarding the “equivocal” range.

## Figures and Tables

**Table 1:** Patient characterization.

Patient Characterization	Cohort (*n* = 135)	Bacterial Infection (*n* = 29)	Viral Infection (*n* = 106)	*p*-Value
Age (years), median (IQR)	3.5 (1.3–6.0)	6.0 (2.5–7.0)	3.0 (1.0–6.0)	<0.001
Male, *n* (%)	80 (59.3)	13 (44.8)	67 (63.2)	0.074
Days from fever onset, median (IQR)	2 (1.0–3.3)	2.0 (1.0–3.0)	2.0 (1.0–4.0)	0.760
Hospitalization duration (days), median (IQR)	3.5 (2.0–6.0)	5.0 (3.0–10.0)	3.0 (2.0–5.0)	0.005
Antibiotic treatment, *n* (%)	61 (45.2)	29 (100.0)	32 (30.2)	<0.001
WBCs(×10^3^/μL), median (IQR)	11,380 (8220–16,270)	14,760 (11,195–22,217.5)	11,000 (7780–15,000)	0.001
ANC (×10^3^/uL), median (IQR)	7000 (4260–9804)	11,908 (7154–19,010.8)	5868.1 (3718.1–8593.0)	<0.001
CRPn (mg/dL), median (IQR)	23 (8.0–78)	132.0 (101–207.5)	14.0 (6–31.033)	<0.001
Final diagnosis, *n* (%) LRTI URTI UTI Other	80 (59.3) 36 (26.7) 4 (3) 15 (11.0)	16 (55.0) 5 (17.0) 4 (14.0) 4 (14.0)	64 (60.4) 31 (29.2) 0 (0.0) 11 (10.4)	
**Microorganisms detected through molecular multiplex-nested-PCR**				
Not detected, *n* (%)	23 (17.0)	8 (25.6)	15 (14.2)	
Viruses, *n* (%) *Human rhinovirus/enterovirus * RSV *Influenza virus* *Adenovirus* *Coronavirus* *Parainfluenza* *Metapneumovirus*	109 (80.7) 54 (49.5) 32 (29.4) 26 (23.9) 19 (17.4) 16 (14.7) 8 (7.3) 1 (0.9)	19 (65.5) 11 (37.9) 1 (3.4) 1 (3.4)	90 (84.9) 43 (40.6) 31 (29.2) 25 (23.6) 19 (17.9) 16 (15.1) 8 (7.5) 1 (0.9)	
Bacteria, *n* (%) *Chlamydia pneumoniae* *Haemophilus influenzae* *Streptococcus pneumoniae*	3 (2.2) 1 (33.3) 1 (33.3) 1 (33.3)	3 (2.2) 1 (33.3) 1 (33.3) 1 (33.3)	0 (0.0)	

IQR, interquartile range; WBCs, white blood cells; ANC, absolute neutrophil count; CRPn, C-reactive protein by nephelometry; LRTI, lower respiratory tract infection; URTI, upper respiratory tract infection; UTI, urinary tract infection.

**Table 2:** BV score comparison to routine biomarkers.

Score		Sensitivity % (95% CI)	Specificity % (95% CI)	PPV% (95% CI)	NPV% (95% CI)	Equivocal
BV	<35 or >65	96.2 (80.4–99.9)	88.7 (80.6–94.2)	69.4 (56.5–79.9)	98.9 (92.6–99.8)	8.9%
	60	93.1 (77.2–99.2)	88.7 (81.1–94.0)	69.2 (56.7–79.5)	97.9 (92.5–99.5)	
CRPn (mg/L)	>60	93.1 (77.2–99.2)	83.0 (74.5–89.6)	60 (49.3–69.8)	97.8 (92.0–99.4)	
	>80	86.2 (68.3–96.1)	94.3 (88.1–97.9)	80.6 (65.4–90.2)	96.2 (91.0–98.4)	
WBCs (×10^3^/μL)	>15,000	50.0 (29.9–70.1)	75.8 (65.7–84.2)	37.1 (25.8–50.1)	84.2 (78.0–88.8)	
ANC (×10^3^/μL)	>10,000	57.7 (36.9–76.7)	85.4 (76.3–92.0)	53.6 (38.8–67.8)	87.4 (81.4–91.6)	

CRPn, C-reactive protein by nephelometry; WBCs, white blood cells; ANC, absolute neutrophil count.

## Data Availability

The data presented in this study are available on request from the corresponding author due to ethical reasons.

## Abbreviations

The following abbreviations are used in this manuscript:

TRAIL	tumor necrosis factor-related apoptosis-inducing ligand
IP-10	interferon gamma-induced protein-10
CRP	C-reactive protein
ED	emergency department
PCR	polymerase chain reaction
WBCs	white blood cells
ANC	absolute neutrophil count
CIs	confidence intervals
SD	standard deviation
IQR	interquartile range
AUC	area under the curve
PPV	positive predictive value
NPV	negative predictive value
